# Special Problems Of Pacing In Children

**Published:** 2003-01-01

**Authors:** Herwig Antretter, Joshua Colvin, Ulli Schweigmann, Herbert Hangler, Herwig Hofer, Karin Dunst, Josef Margreiter, Guenther Laufer

**Affiliations:** 1 Department of Cardiac Surgery; 2 Department of Pediatric Cardiology; 3 Anesthesiology and Intensive Care Medicine; University Hospital Innsbruck, The Leopold-Franzens-University Innsbruck, Austria, Europe

**Keywords:** pediatric cardiac pacing, epicardial pacing, transatrial pacing, growth

## Abstract

The number of children suffering from congenital or acquired rhythm disorders, and therefore being pacemaker dependent, is very small. This is one of the reasons why a special hardware has never been developed for this cohort.

Pacemaker implantation into children does not differ substantially from operations in adults. But there are several important points which have to be fulfilled in these small patients in order to guarantee a complication free function. As most of these children remain pacemaker dependent a lifetime, it is of tremendous importance to minimize all revisions regarding the implanted systems and to enable our small patients a high and therefore nearly normal quality of life.

Pros and cons of different surgical approaches, implantation sites and the problem of growth after pacemaker implantation in children are considered.

## Introduction

 There are no substantial differences regarding the surgical procedure of pacemaker implantation in children and adults. Nevertheless pacing in children needs a more differentiated assessment. Pacemaker implantation in newborns and children requires a highly experienced and skilled surgeon, who is able to evaluate the problems in these small patients and to estimate all consequences of a lifelong electric stimulation of the heart,  often lasting several decades.

The number of children who need a pacemaker (PM) is small and negligible in relation to the number of adults being supplied with a PM. Only about one percent of all pacemakers are implanted into children [1]. For this reason no hardware was specifically designed for this small cohort and up to this day all implantations in children are carried out with the hardware which was solely developed for the grown-up population.

In the last four decades since the first human pacemaker implantation by Senning [2] all components of this therapy have undergone such rapid technological development that most types can also be implanted in very small patients without creating remarkable problems. Above all this has led to a continuous reduction in the hardware size, which now routinely allows the implantation of physiological dual chamber pacing systems even in infants.

##  Indications

 Indications for pacing in newborns and infants are devided predominantly into three groups: congenital abnormalities of the conduction system, acquired heart blocks after cardiac surgery for correction of congenital defects and sinus node diseases. Rare indications include the therapy of tachyarrhythmias [3], of hypertrophic obstructive cardiomyopathy [4,5] and of the long-QT-syndrom [6]. The precise indications are available in the guidelines of the American College of Cardiology/American Heart Association Task Force on Assessment of Diagnostic and Therapeutic Cardiovascular Procedures for implantation of cardiac pacemakers in children [7].

## Implantation

 In contrast to adults implantation of a pacemaker in infants always requires the individual assessment of access (endovenous versus epicardial), of the leads in use, of the implantation site (infraclavicular versus abdominell - or alternative implantation places) and of the topography (subcutaneous versus submuscular). Also the expected growth of the child has to be taken into consideration during implantation. And finally the lifelong dependency on pacing therapy and the many future revisions therefore have to be considered.

## Choice of access and pacing leads

### Transvenous versus epicardial access

**Figure 1:**
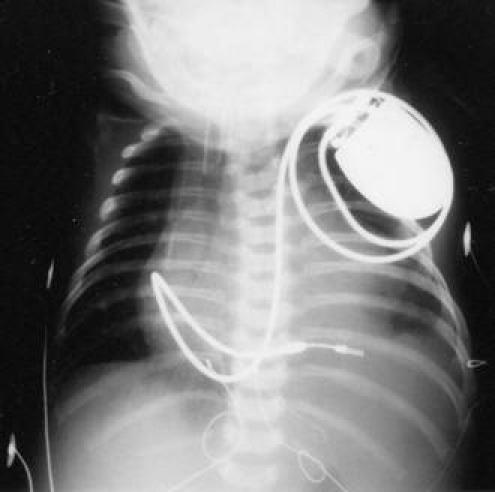
Transvenous pacing system in a 2990 gram and 47 cm long newborn via left superior vena cava to right ventricle (bipolar steroid eluting Medtronic screw in 4068-52cm) with the pulse generator (Medtronic MicroMinix 8360) in a subcutaneous left pectoral pouch (Although this operation was carried out by one of the authors (H.A.) in 1993, we are today no longer able to explain, why the generator was not implanted submuscularly in this case).

Currently it is recommended to use the epicardial approach in infants until the age of 3 to 4 years in order to prevent a lesion of the subclavian vein [8]. However we could demonstrate a complication free endovenous approach in a newborn suffering from anatomical vascular abnormalities [9] (Figure 1). Finally the conventional endovenous PM approach - as practised in adults - is a smaller burden for children than an epicardial lead positioning after (partial) sternotomy or left sided thoracotomy.

A 7 French bipolar pacing lead can be implanted in nearly every mature newborn after direct puncture of the subclavian vein or under direct vision after freeing the vessel, provided that one is able to work with adequate surgical attention and exactness. Advancement of the electrode and  positioning at the desired place (atrial or ventricular) is possible without difficulties. The trend from epicardial towards endovenous leads has been steadily increasing over the years [10]. But on the other hand there are also implanters who recommend a transvenous approach via the internal jugular vein [11] in children under the age of three. 

Lead refinement allowes further reduction in the diameter of endovenous, bipolar, active screw-in electrodes [12], so that we can expect fewer vein complications during long term follow-up (especially stenosis or thrombotic occlusion) in the future.

Nevertheless lead revisions due to growth of the afflicted children remains a considerable problem in pacing therapy until puberty. Fibrotic attachment to the vessel wall (mostly in the confluens area of the subclavian and brachiocephalic vein with the superior caval vein) can compromise a later advancement of the lead. This results in implantation of an additional new lead in a relatively short period of time after the first implantation (in spite of the fact that the first electrode shows perfect electrical values!). On the other hand, if one takes this problem into consideration and gives the lead more redundancy at the time of implantation (for example a lead loop in the right atrium) to prevent lead displacement caused by growth as long as possible [13], a higher displacement rate has to be accepted due to tension the floating loop exerts on the tip of the endocardial electrode. Besides, surplus lead material might be dislocated into the right ventricle triggering arrhythmias. Or it might be displaced through the right ventricular outflow tract into the pulmonary trunk, thus resulting in a hemodynamically relevant pulmonary valve insufficiancy.

Gheissari et al. [14] have calculated that a 80 millimeter right atrial lead loop allows 6-12 years growing in children with a mean of eight years without neccessitating a reoperation for lead adjustment (that means every year approximately 10 millimeters of lead length are necessary to compensate body growth).

**Figure 2:**
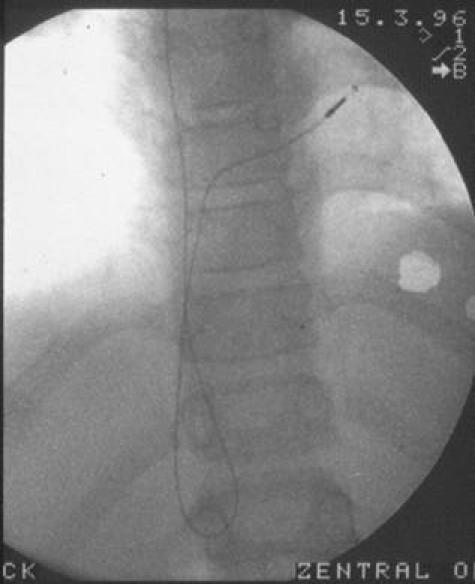
Posteroanterior radiography immediately before reintervention showing the inferior vena caval loop of the pacemaker lead strongly attached to the endothelium. The tip of the electrode is still attached the right ventricular wall. At that time an exit block was predominantly existent.

Gasparini et al. [15] suggested to leave a redundant lead loop within the inferior vena cava (IVC) in order to allow further growth by shortening the excess loop. Unfortunately we had contrary experiences in a pacemaker dependent child: the electrode got firmly attached to the endothelium of the IVC, thus the expected lead release failed causing an exit block and making an emergency admission with rapid revision due to of intermittent loss of capture necessary [16] (Figure 2).

Some other authors suggest lead fixation at the site of venous entrance with slowly absorbable sutures as a solution to the growth problem [17]. An absorbable Dexon suture keeps the lead in place after implantation until fibrotic tissue encapsulates the lead body. Finally, after resorption of the absorbable suture spontaneous lead migration through the "endothelial sheath" is possible - corresponding to the child's growth. This "sliding technique" normally does not work as mentioned above, because the endovenous part of the lead is not only fibrotically fixed at the tip but is also often firmly attached to the endothelium of the large venous vessels. Additionally the extravasal lead excess, which often is long in standard leads and therefore has to be coiled behind the generator, is fixed tightly to the wall of the pocket most of the time [18]. Therefore it is more a well meant idea than the reality that the lead will, over the years, migrate centimeter by centimeter into the vein, proportional to the child's growth [19].

 As children will mostly need lifelong pacing therapy they will consume several leads  during a life-span. Therefore we only implant steroid eluting screw-in leads with an isodiametric lead body during infancy and adolescence. The isodiametric construction allows an easier and safer elective extraction [8,20], which we always try to carry out if a second new lead has to be implanted [21]. Besides, active fixation enables an anchorage at every desired position (also in the right chamber), which can be important in anatomic variations or complex cardiac malformations. Continuous release of steroid in the first phase after implantation ensures stable low chronic stimulation thresholds [22] and is available today in most of the modern screw-in models.

 Severe tricuspid regurgitation after pacemaker lead implantation is a rare complication but it can cause dramatic clinical situations [23]. After endovenous lead implantation also one has to take the possibility of perforation into consideration, analogous to adults.  Even temporary endovenous cardiac pacing can cause atrial or ventricular perforation [24].

 If endovenous implantation of leads in a child is impossible because of severe obstruction or occlusion of the subclavian vein or the superior vena cava (often after cardiosurgical procedures) one should think about an interventional therapy combined with surgical treatment before planing an epicardial or alternative approach. Vessel recanalisation can be achieved with ballon dilation and subsequent stent implantation [25]. Through the vein secured in this way a transvenous pacing lead that permits further endovenous pacing can be inserted immediately or some (6-8) weeks later.

Primary indications for epicardial pacing in newborns or infants are:

1. Impossibility of introducing a lead into the venous system (surgical reasons: for example: baby too small).
2. Venous abnormalities or congenital malformations which make a venous lead implantation impossible: either inborn (discordant atrioventricular connection, tricuspid atresia) or acquired (after open heart surgery: total cavopulmonary connection - Fontan circulation).
3. After surgical correction of complex cardiac malformations which require additional heart operations with the use of extracorporal circulation (heart lung machine).
4. If all upper venous vessels have thrombotic occlusions without the possibility of interventional reopening and if alternative approaches are not possible [26].
5. If a right-to-left shunt with the risk of systemic embolisation exists [27].
6. If one wants to prevent the endovenous problems regarding growth during the first years of life in a totally pacemaker dependent child as described above.

Different solutions exist for epicardial lead fixation, regardless whether a cork-screw mechanism or only a suture fixed epicardial system is used. If possible, these electrodes should be steroid eluting and bipolar. Currently excellent bipolar steroid eluting epicardial systems are commercially available. They can prevent one of the most limiting complications of older epicardial leads: chronic high thresholds resulting in premature battery depletion and also a permanently threatening exit block [28]. Goldman Cutler [29] could demonstrate a stable low pacing threshold with unipolar, epicardial steroid-eluting pacing. In a recent publication Beaufort-Krol [30] reported nearly equally good pacing and sensing thresholds for steroid-eluting epicardial pacing leads, similar to conventional endocardial pacing leads in children. When combined with the technique of automatic ongoing capture verification by evoked response signal detection and automatic ongoing output adjustment (autocaptureÒ function), it is possible to save battery current substantially, even in epicardial lead systems without any hazards regarding stimulation safety, and therefore extend the battery life markedly [31].

One additional disadvantage of epicardial pacing leads is a slightly higher fracture rate because of the higher mechanical stress compared to endovenous leads [32].

An interesting alternative for endocardial pacing is the ***transatrial approach*** [26,33,34]. Following a anterolateral, rightsided thoracotomy (5^th^ or 6^th^ intercostal space) or a median sternotomy, pacing leads are directly implanted through the right atrium and subsequently positioned endovenously into the right atrium or right ventricle. Indications for this more invasive approach are occluded or obstructed central venous vessels, hypoplastic central veins or if there is no connection between the vena cava superior and the right atrium (due to of complex vascular or cardiac malformations) or if the passage between the superior vena cava and the right atrium was disconnected surgically (Fontan-circulation, Glenn shunt). A transatrial approach can replace the epicardial stimulation, which would normally be used in these situations and prevents its potential complications.

The major disadvantage - like in many alternative surgical approaches - is the complexity of repeated revisions in postoperative complications (for example lead dislodgement).

**Figure 3:**
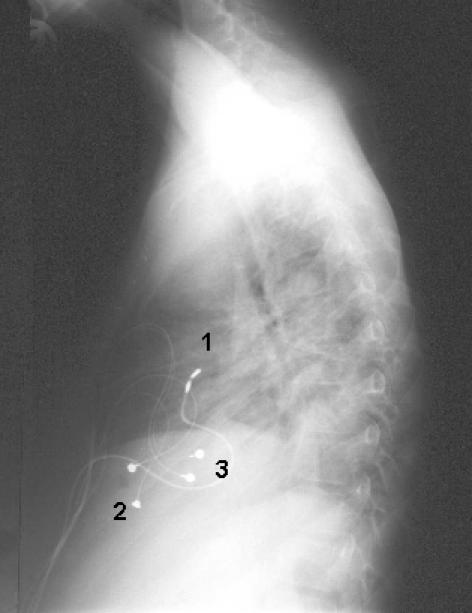
Transatrial lead implantation in a 12 year old boy with complex cardiac malformations. He had several open heart procedures and finally in 1995 a modified Fontan procedure with the addition of a fenestrated atrial baffle was accomplished. Sick sinus syndrome with bradycardia and dizziness was diagnosed in 2001. Because of the caval pulmonary connection a venous approach for lead insertion from cranial was impossible. Therefore, after median sternotomy a bipolar screw-in lead was implanted transatrially into the venous system (1) together with a right ventricular epicardial bipolar, steroid-eluting epicardial electrode (2). The pacemaker was programmed to DDDR mode, with the AV-conduction time so long that  intermittent atrial pacemacer stimulation allowed physiologic stimulation of the ventricle. As an atrial back up lead a second epicardial bipolar steroid-eluting electrode was placed on the right atrium (3) with the lead body tunnelled to the generator pocket. In case of transatrial lead failure (e.g. exit block), revision would not neccessitate a rethoracotomy, but only an incision into the abdominal generator pocket and the exchange of the epicardial atrial lead.

After a child with extremely complex cardiac malformations and a history of several open heart procedures developed pacemaker dependency due to sick sinus syndrome, we opted for the transatrial approach to prevent resternotomy.  Besides implanting the transatrial electrode for atrial pacing, two steroid eluting bipolar epicardial pacing leads for atrial as well as ventricular stimulation were implanted parallel and all the leads were tunneled to the abdominal generator pocket (Figure 3).  If, in the remote future, the transatrial lead stimulation fails, only the abdominal pocket has to be reopened with a relatively small incision and the transatrial lead has to be exchanged for the epicardial one without the need for resternotomy or rethoracotomy. At present the child is stimulated transatrially in the physiologic rate adaptive AV mode, naturally with a very long programmed AV-conduction time to allow physiological excitement of the right ventricle.

The transatrial approach offers another advantage: the growth related difficulties in children with endovenous leads are minimized. By using the transatrial approach one only has to consider the growth of the heart to calculate the additionally required length of the lead. Redundant lead loops or later revisions to reposition a lead by a few centimeters in order to prevent loss of capture due to growth, are of little concern in this access.

### The generator pocket in children

In order to prevent pocket related problems in small children we implant all generators subpectorally (submuscularly) a priori. This procedure strictly requires bipolar leads to prevent pectoral muscle convulsions. Today bipolar leads are so flexible and thin that transvenous insertion and correct positioning is possible in nearly every small infant. In adults we also generally only use bipolar leads in our hospital.

We always chose the submuscular placement of the pulse generator in our pediatric pacemaker population and as yet were never confronted with pocket related complications [35], in particular erosions or thinning of the skin. Additionally the cosmetic aspect is much more favorable which is a very important aspect for the younger population [36]. Especially during puberty and adolescence when children are forming their body image and identity, there are remarkable conflicts regarding the acceptance of these lifesaving implants and this may have psychosocial implications  (inferiority complex; anxiety about being teased, etc.) [1,37].

Additionally the subpectoral pocket can also prevent rare complications in children such as the Twiddler syndrome [38], a situation which was observed predominantly in adults.

**Figure 4:**
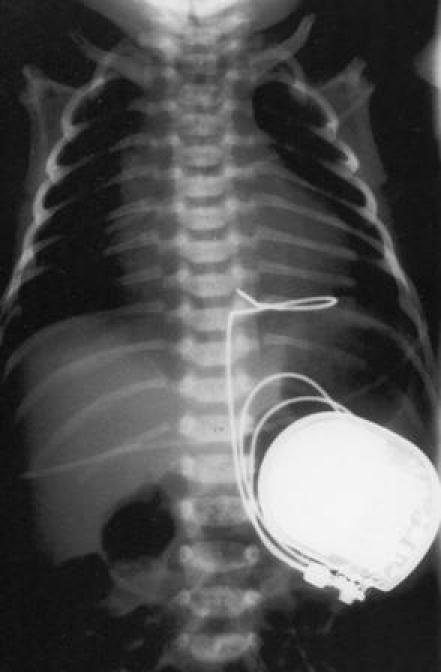
Pulse generator placed in the abdominal wall (into the rectus sheath) in a 3 day old boy, 2700 grams, with congenital complete heart block. As this implantation was carried out in 1993, a unipolar epicardial lead was used (Osypka MP 47).

 By using epicardial systems, which are implanted only in babies, mostly by a subxiphoidal approach, a generator pocket is created abdominally behind the anterior sheet of the rectus muscle (Figure 4). With this procedure the visual impression is also acceptable and we have never encountered noteworthy pocket complications.

 Therefore we would recommend never to implant a pulse generator in a child subcutaneously but always in a submuscular location, and this not only in the pectoral region but also in the abdominal wall.

Several years ago the size of a pulse generator was a true problem in infants [36] but today even sensor driven dual chamber pulse generator are so small and light that even implantation in newborns is feasible without problems. Therefore the smaller the patient, the more important the selection of the pulse generator model regarding the upper tracking rate. In a newborn, sometimes upper frequencies of 150 stimuli per minute or more are necessary and not every modern pacemaker can be programmed permanently with this upper rate!

## VDD pacing in children

 When the single pass lead was implemented into clinical practice for VDD pacing in complete heart block, only a few case reports described its insertion into children. Meanwhile larger series have been published and more experiences have been made with the pediatric cohort [39-41]. Certainly most of these patients are larger children, even adolescents. In the cited publications mean ages were 10.1, 7.9 and 9.9 years! Insertion of a VDD lead into a child is no problem for an experienced surgeon and over a longer period of time maintenance of adequate atrial signals with reliable pacing is possible, despite the considerable growth of some children [40,41].

So far we did not use this stimulation mode in children, because presently no isodiametric VDD lead with a screw-in mechanism is commercially available. Furthermore at present all distributable VDD leads are thicker than bipolar single chamber standard leads. During growth the exact same problems can arise with VDD leads as we sufficiently know from bipolar leads in single or dual chamber pacing. This means that also when using a VDD system the lead has to be pushed forward as the child continues to grow, possibly even more often because the atrial sensing rings could lose the capability of detecting reliable atrial amplitudes after gradually changing their positions out of the right atrium in the direction of the superior vena cava. (Intermittent) Loss of sensing of an adequate atrial amplitude results in a failure of the physiological rate responsive AV synchronous pacing and also reqiures revision of the implanted system. For the sake of completeness it should be mentioned that every VDD pacemaker has an automatic sensor driven rate adaptive ventricular pacing mode incorporated, which permits activity operated ventricular stimulation until the next revision will be carried out. 

## Life long pacemaker dependency

As pacemaker dependent children require lifelong electrical therapy and consequently are inseparable from the implanted hardware, some special points regarding surgical interventions have to be considered. These have become standard procedures in our University Hospital Innsbruck:

Site of implantation should be conserved as long as possible. If one switches too quickly to another implantation site or to the contralateral side, the number of untouched potential places of implantation and intact venous access routes are understandably reduced. In the remote future, after decades of pacing therapy this can generate remarkable problems.
Thus pediatric pacing therapy should only be carried out by experienced surgeons, well equipped with routine in this therapy that act with essentially required cautiousness and are able to prevent or to minimize complications [42,43].
Prior to every single lead insertion we sonographically judge the central venous access in terms of stenosis or occlusion. Dependent on the result of this examination we try to plan the operation (simple implantation versus interventional approach with dilatation, stenting with subsequent surgery or alternative access - as mentioned above).  As many of these kids have previously had open heart surgery or central venous catheters they are predisposed for venous obstructions and occlusion.
If a new pacing lead has to be implanted endovenously, the old screw-in lead is always extracted during the same session (if necessary with extraction kits).
The dimension of further growth and the need for an additional endovenous lead loop has to be considered preoperatively.
In the pediatric population we only implant the latest rate adaptive pacemakers intending to reach the most physiological pacing mode as soon as possible. State of the art pacemakers additionally offer several important diagnostic tools.

Recent publications have pointed out the problem of left ventricular systolic as well as diastolic dysfunction after long-term right ventricular apical pacing in the young [44]. Unphysiologic apical stimulation does not only result in a paradoxical septal wall motion but it also leads to an impaired left ventricular contraction and a global and regional reduced LV function. Pathological changes were not only detected histologically but also on cellular levels [45]. The unphysiologic, asynchronous patterns of right and left ventricular contraction with right ventricular (endovenous or epicardial) apex affixed leads can be reduced by stimulation from the septal and high right ventricular outflow tract. With these positions echocardiographic findings show markedly normalized ventricular contraction patterns.

 It has to be our primary goal to enable pacemaker dependent children a life as normal as possible. The pediatric cardiologist has to assure this with individually tailored follow-ups after pacemaker implantation. The surgeon however should make certain that the amount of future interventions- and there will be many of them in the life of these young patients until they reach old age - are reduced to an absolute essential minimum and carried out with extraordinary quality and perfect results.
